# Dovramilast for Erythema Nodosum Leprosum in Patients With Leprosy in Nepal: Protocol for a Phase 2 Open-Label Pilot Study

**DOI:** 10.2196/88069

**Published:** 2026-06-17

**Authors:** Mahesh Shah, Divya RSJB Rana, Kapil Dev Neupane, Preeti Maharjan, Suwash Baral, Reejana Shrestha, Jivan Shakya, Binod Aryal, Bishwanath Acharya, Indra Bahadur Napit, Deanna A Hagge

**Affiliations:** 1 Department of Dermatology, Anandaban Hospital, The Leprosy Mission Nepal, Godawari-06, Tikabhariav, Lalitpur, 151, Nepal, 977 9841203417; 2 Research Department, Anandaban Hospital, The Leprosy Mission Nepal, Lalitpur, Nepal; 3 Zero Leprosy Pathfinder Consulting, Baton Rouge, LA, United States

**Keywords:** CC-11050, clinical trial, dovramilast, erythema nodosum leprosum, ENL severity scale, leprosy, Nepal

## Abstract

**Background:**

Erythema nodosum leprosum (ENL) is an immunological complication affecting up to 10% of borderline lepromatous and 50% of lepromatous leprosy cases with a high bacterial index (3‐6+). Current treatments often require prolonged therapy over years and cause significant long-term side effects, highlighting the need for new therapies. Therefore, it is imperative to identify new therapies for ENL. Phosphodiesterase 4 inhibitors are a class of compounds that mediate immune homeostasis and have the potential to treat ENL with fewer side effects. Dovramilast (formerly CC-11050) is an anti-inflammatory phosphodiesterase 4 inhibitor; to date, it has been shown to be well tolerated in phase 1 human studies. This is the first phase 2 study of dovramilast in ENL.

**Objective:**

This phase 2 pilot study aims to assess the safety and tolerability of dovramilast and to explore preliminary clinical responses in patients with new or recurrent ENL.

**Methods:**

This study was designed in two steps: (1) 28 days of treatment for 10 males to ensure safety and (2) an initial 12-week treatment course, with the option to retreat if further ENL episodes occur (up to 48 wk of treatment), for up to 40 males or females, as required on a case-by-case basis. Demographic, clinical, and laboratory data will be collected longitudinally, along with the ENL International Study Group ENL Severity Scale, Brief Pain Inventory, and Douleur Neuropathique 4 questionnaire for the identification of neuropathic pain. Patients will be followed for 12 months for ENL recurrence after cessation of the study drug. Safety will be evaluated through adverse event reporting and laboratory monitoring, while efficacy will be assessed using descriptive and trend-based analyses of changes in ENL severity, pain, and clinical outcomes over time.

**Results:**

The initial safety evaluation (step 1) of this phase 2 pilot study was completed and reviewed by an independent Data Safety Monitoring Board, which did not identify safety concerns that would preclude continuation of the study. Based on the step 1 results, the study proceeded to step 2, which evaluates longer-term administration of dovramilast in male and female participants. As of April 2026, a total of 15 participants have been recruited. All study data are anticipated to be collected by September 2027, with publication of the main trial results expected by December 2027.

**Conclusions:**

This phase 2 pilot study is designed to generate preliminary data on the safety, tolerability, and potential clinical signals of dovramilast in patients with new or recurrent ENL. Findings from this exploratory trial will inform the feasibility, outcome measures, and design of future adequately powered randomized controlled studies and do not directly support changes to current clinical practice at this stage.

## Introduction

### Leprosy and Leprosy Reactions

Leprosy is an important but neglected global disease. Effective multidrug therapy (MDT) has resulted in a reduced prevalence of the disease, and leprosy case detection rates have stagnated over recent decades, as reported by the World Health Organization (WHO) [1,2]. Leprosy is caused by the acid-fast bacteria *Mycobacterium leprae* or *M. lepromatosis* and primarily affects the skin and peripheral nerves [3,4].

In 2023, 72.9% of new leprosy cases detected were multibacillary [1] presenting with one or more cardinal signs, including greater than 5 lesions, detectable *M. leprae* bacilli, or peripheral nerve involvement, compared to paucibacillary presenting with less than 5 lesions and no detectable bacilli [5]. Delayed diagnosis or treatment of leprosy can lead to permanent nerve damage and disabling deformities. Neuropathy may develop before, during, or long after treatment due to persistent mycobacterial antigens that drive immunological reactions and accelerate nerve injury. Consequently, leprosy reactions are a major contributor to long-term disability in affected individuals [6-12].

Two major types of leprosy reactions occur, affecting up to 30% to 50% of all leprosy cases at some point: type 1 reaction (T1R, reversal reaction) and erythema nodosum leprosum (ENL, also called type 2 reaction) [7,13-15]. T1R typically involves increased cell-mediated immunity toward leprosy antigens and most often develops in borderline tuberculoid, borderline borderline, and borderline lepromatous (BL) cases. However, ENL develops only in BL and lepromatous leprosy (LL) cases, with high bacterial loads associated with reduced T cells and increased antibody responses to leprosy antigens. ENL may occur before the start of leprosy treatment but can also develop during or years after MDT [6,7]. After treatment for leprosy, people may experience episodes of ENL for years because mycobacterial antigens persist despite bactericidal MDT [6-8,16]. Neuritis can occur either alone or in combination with T1R or ENL. Some patients experience reaction cases typical of T1R and ENL in succession or in combination [17,18].

### Characteristics of ENL

ENL presents as new, red, painful nodules, usually on the legs, arms, face, and sometimes on the trunk, and it varies in severity [8,19-22]. It is characterized by the rapid appearance of successive crops of painful and erythematous subcutaneous nodules that may ulcerate [19]. ENL may be present at the time of leprosy diagnosis or develop within the first few years after starting MDT [6-8].

Individual ENL episodes can last months or years, with a significant proportion of patients experiencing recurrent episodes [6,8]. Recurrent ENL reactions are defined as more than 1 ENL episode with the same characteristics as acute ENL. Chronic ENL is defined as an episode lasting for more than 6 months of treatment. The main risk factors for ENL are LL or BL leprosy with high bacterial load and a bacterial index greater than or equal to 3+ [11,19,23]. Of these, 10% of patients with BL and 50% of patients with LL develop the first ENL episode coincident with leprosy diagnosis, during MDT, or in the years after MDT completion. Other less well-defined factors that increase risk include pregnancy, lactation, puberty, intercurrent infection, vaccination, and stress [16].

**Figure 1.**
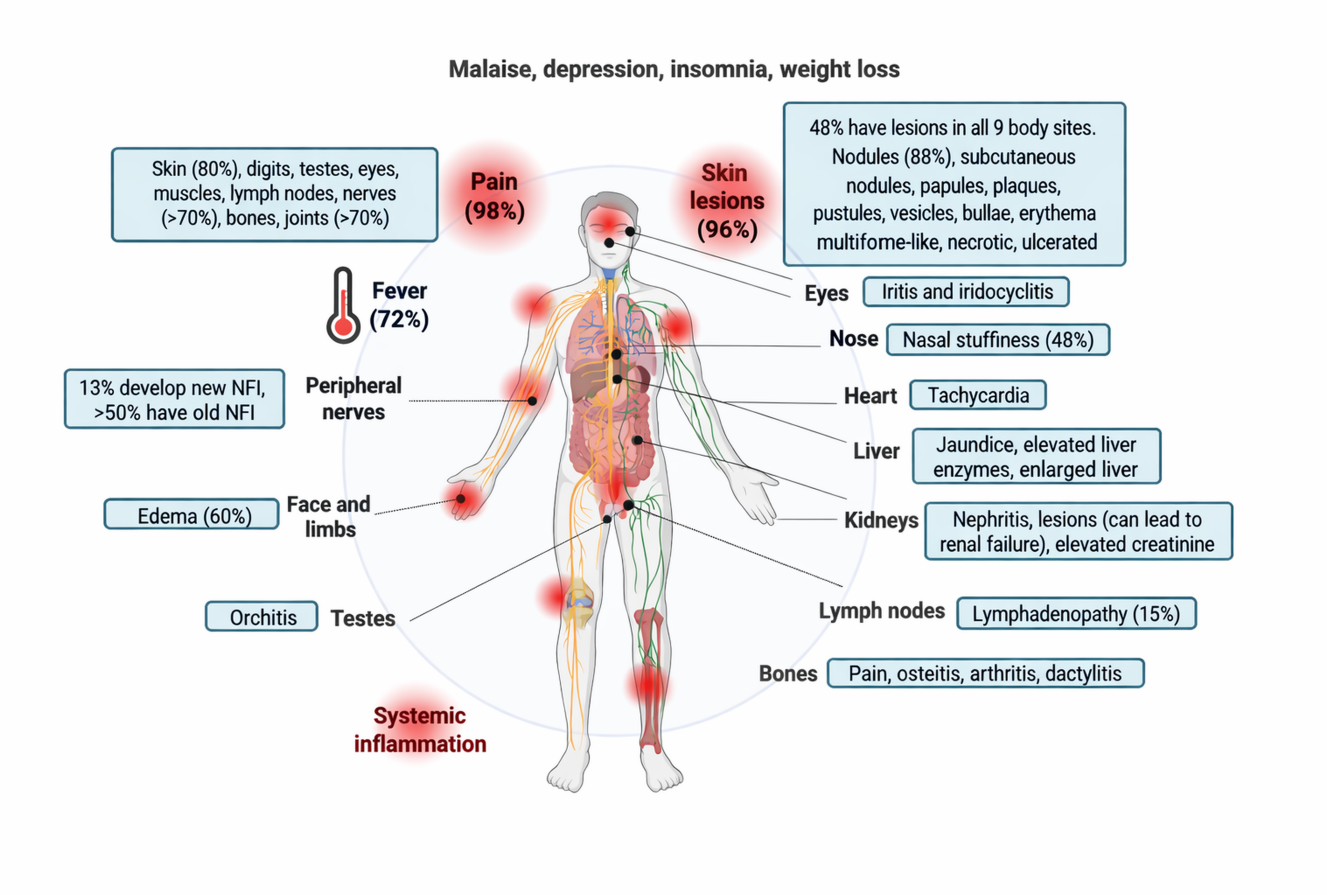
Symptoms and organ involvement associated with erythema nodosum leprosum (ENL). The peripheral nervous system is shown on the left and the lymphatic system on the right. Symptoms and incidence are consolidated from reviews and ENL cohort reports [19,25]. Created in BioRender by author DAH [26].

Systemic signs and symptoms, such as fever, anorexia, and malaise, signal its occurrence; however, these symptoms are not always recognized in association with leprosy and can be initially misdiagnosed [19]. See Figure 1 for symptoms and organ systems that are variably associated with ENL. The ENL International Study Group ENL Severity Scale (EESS) focuses on a subset of 10 major symptoms as severity indicators common to both males and females: pain, fever, number of ENL lesions, inflammation of ENL lesions, extent of ENL lesions, peripheral edema, bone pain, inflammation of joints and/or digits, lymphadenopathy, and nerve tenderness [19,24].

When the reaction is mild, only the skin is affected, often in combination with a low-grade fever. When the reaction is severe, the nodules are multiple and may ulcerate, there may be a high fever, and other organs such as the nerves, eyes, joints, testes, and lymph nodes may be inflamed. Typically, recurrence in these patients presents with the emergence of red subcutaneous swelling, often associated with fever and pain.

Apart from disability and stigma, ENL can cause devastating psychosocial, physical, and economic hardship [9,27,28] and multiple hospitalizations in affected patients. Moreover, the side effects of treatments create vulnerabilities to other diseases, prevent patients from leading normal social or emotional lives or engaging with others, cause loss of employment or cessation of schooling, or degrade their enthusiasm to struggle against these obstacles.

### Potential Causes of ENL

The pathogenesis of ENL is poorly understood [14,29,30]. ENL was previously considered to be an immune complex–mediated phenomenon; however, very limited and sometimes contradictory evidence is available to support this [31].

As is common in BL or LL cases, studies have indicated that ENL patients have high levels of anti*–M. leprae* antibodies, as well as persistent levels of *M. leprae* antigens. The most characteristic feature of the lesions is the presence of a neutrophilic infiltrate. Immunoglobulin and complement deposition are present in the skin lesions. Serum complement levels are decreased, and the expression of interleukin (IL)-6, IL-8, IL-10, IL-4, and IL-5 is increased. Activation of both toll-like receptor 2 and fragment crystallizable receptors has been shown to induce IL-1β, which upregulates the expression of E-selectin and neutrophil binding in endothelial cells. Additionally, neutrophils can be stimulated by *M. leprae* to secrete tumor necrosis factor (TNF)-α and IL-8, thereby increasing inflammation [14,29,30].

Cell-mediated immune responses also contribute to the pathogenesis of ENL. Several studies have supported the association of a T-helper 1-type response with high levels of interferon-gamma and IL-12 [30]. CD4^+^ T cells have been shown to outnumber CD8^+^ T cells by 2 to 1. On the other hand, other studies have suggested that a T-helper 2-type response dominates, with high levels of TNF-α and IL-6 detected in the serum and skin lesions [10,32]. More recently, single nucleotide polymorphisms in the IL-6 gene were found to be associated with ENL [33]. Soil-transmitted helminth–induced immunomodulation across many shared cellular and cytokine pathologies may also be relevant to the development of neuritis, T1R, and ENL reaction episodes in coendemic populations [34].

### Treatment for ENL

Prednisolone, thalidomide, and clofazimine are currently recommended by the WHO as treatment for ENL; however, ENL treatment has been controversial for decades [20,21,28,35,36]. Prednisolone is the most common first-line treatment in most low- or middle-income countries and leprosy-endemic countries (except Brazil, which uses thalidomide). Prednisolone is generally available and affordable, but, as ENL may require years of treatment, patients can develop severe consequences such as diabetes, cardiovascular problems, blindness, and osteoporosis leading to bone fractures [37-39].

Thalidomide, the most recently recommended therapeutic agent for the treatment of ENL, was developed more than 50 years ago and has both immunoregulatory and sedative activity [40]. However, owing to the teratogenicity and related consequences in the 1960s, thalidomide is often restricted or unaffordable for most ENL cases in endemic countries. High-dose clofazimine has anti-inflammatory properties and can be given in combination with prednisolone. Studies, however, have not indicated a significant impact, and patients rarely tolerate clofazimine due to skin pigmentation [41].

Both high-dose prednisolone and thalidomide are sometimes insufficient to control severe refractory ENL [42-44]. Many cases are chronic and experience ENL symptom relapse soon after the decrease in dosage or cessation of drugs [35]. Thus, a safe and effective drug that can be used for a shorter duration and imparts long-term remission has always been envisioned [28]. These problems are compounded by the lack of alternative treatments with established efficacy for ENL. Various efforts have been made to develop alternative ENL treatments, most notably pentoxifylline, azathioprine, cyclosporine, methotrexate, and anti-TNF biologics such as etanercept and infliximab [45-59].

### Dovramilast in Relevance to ENL

**Figure 2.**
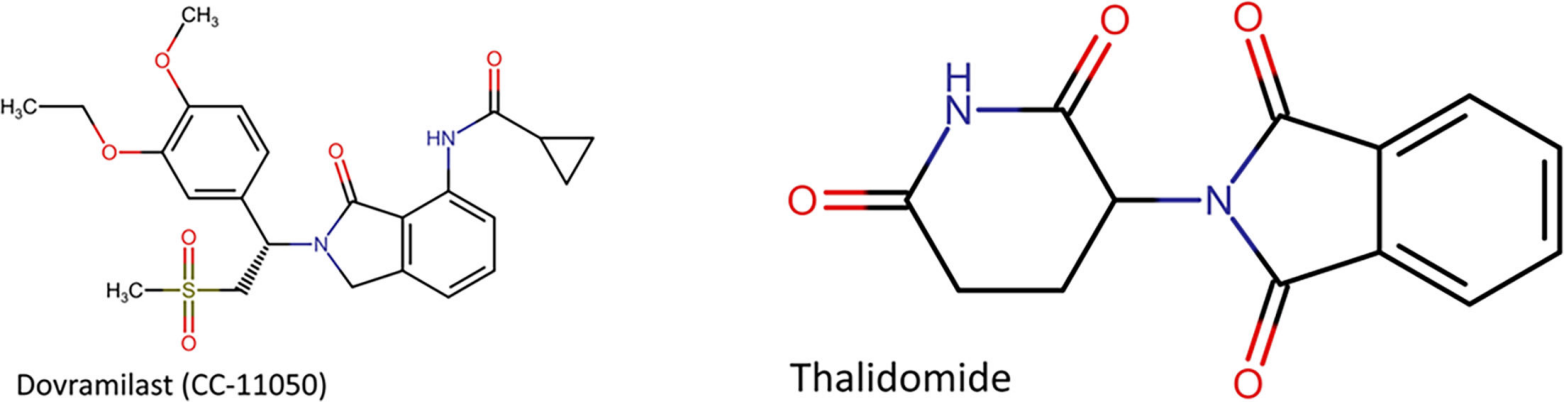
Chemical structures of dovramilast and thalidomide. Figures were drawn using the RCSB PDB Chemical Sketch Tool with chemical Simplified Molecular-Input Line-Entry System (SMILES) from PubChem.

Dovramilast has been evaluated as an adjunct host-directed therapy in HIV, tuberculosis, and SARS-CoV-2 infection models and clinical studies, demonstrating immunomodulatory effects and a favorable safety profile [60-67]. Preclinical studies in rabbit and mouse models of tuberculosis showed improved bacterial clearance and reduced lung inflammation and fibrosis. Phase 1 and phase 2 clinical trials in HIV and tuberculosis patients confirmed good tolerability with no serious adverse events (AEs) and evidence of improved clinical outcomes [60,64,67]. Although previously studied as an adjunct host-directed therapy in infectious diseases, ENL represents a distinct immunological complication, supporting the rationale for evaluating dovramilast as a novel therapeutic option distinct from thalidomide [36,68,69]. The structural difference between dovramilast and thalidomide is presented in Figure 2.

Therefore, the primary objective of this phase 2, open-label clinical trial is to evaluate the safety and preliminary efficacy of dovramilast in adults with new or recurrent ENL. The primary research question is whether dovramilast is safe and well tolerated when administered for short- and longer-term treatment cycles in patients with ENL. Secondary research questions address whether dovramilast treatment is associated with improvements in ENL severity, pain, and clinical manifestations over time, and whether repeated treatment cycles can reduce recurrence without the need for prolonged corticosteroid or thalidomide use. We hypothesize that dovramilast will demonstrate a favorable safety profile and lead to clinically meaningful reductions in ENL severity and symptom burden, supporting its further evaluation as an alternative therapeutic option for ENL.

This protocol is written based on SPIRIT (Standard Protocol Items: Recommendations for Interventional Trials) guidelines (Checklist 1) [70].

## Methods

### Research Question and Hypothesis

The primary research question of this phase 2 pilot study is whether dovramilast is safe and well tolerated in patients with new or recurrent ENL. Secondary research questions explore whether dovramilast treatment is associated with improvements in ENL severity, pain, and clinical manifestations over time and whether repeated treatment cycles may reduce recurrence. Given the exploratory nature of this pilot study, formal hypothesis testing is not planned; instead, the study is designed to generate preliminary safety, feasibility, and clinical trend data to inform future randomized controlled trials.

### Aims and Objectives

The primary objective of this study is to evaluate the safety and tolerability of short- and longer-term administration of dovramilast in adults with new or recurrent ENL. Secondary objectives include assessing preliminary clinical signals of efficacy, including changes in ENL severity, pain, and neurological symptoms, as well as documenting patterns of remission and recurrence. Exploratory objectives include evaluating biological correlations of treatment response using plasma and skin biopsy samples to support mechanistic understanding.

### Study Setting and Sites

The study will be conducted at Anandaban Hospital, The Leprosy Mission Nepal, located in Lalitpur District, Nepal, along with its affiliated satellite clinics. Anandaban Hospital is a national referral center for leprosy and leprosy-related complications and provides specialized clinical and research infrastructure for ENL management and follow-up.

### Sample Size and Justification

The planned sample size includes 10 male participants in step 1 and up to 40 additional male and female participants in step 2. Anandaban Hospital is a tertiary referral center for the management of leprosy reactions and related complications, with approximately 60 to 70 patients with ENL presenting annually. A target sample size of 40 participants was selected based on feasibility considerations, recognizing that not all presenting patients would meet the study’s inclusion criteria or be eligible for enrollment.

Moreover, as this is an observational pilot study, no formal statistical power calculation was performed. The selected sample size is intended to allow assessment of feasibility, safety, and variability in clinical outcomes and to generate preliminary evidence to inform the design and sample size estimation of a future multicenter randomized controlled trial.

### ENL Case Definition

ENL definitions are aligned with the ENL International Study Group definitions [19] and the EESS [24]. ENL is defined as occurring when an individual diagnosed with BL or LL leprosy and a bacterial index >3+ develops 10 or more tender papular or nodular or both types of skin lesions. Other ENL symptoms may also be included but not be limited to those listed in the EESS.

There are 3 clinical patterns by which ENL is described [24]:

1. Acute for a single episode lasting less than 24 weeks
2. Recurrent if a patient experienced a second or subsequent episode of ENL occurring 28 days or more after stopping treatment for ENL
3. Chronic if occurring for 24 weeks or more during which a patient has required ENL treatment either continuously or where any treatment-free period had been 27 days or less

This initial phase 2 clinical trial will focus on acute and recurrent forms of ENL.

The EESS [24] will be used to establish the presence and severity of ENL symptoms during the trial.

### Study Design

#### Overview

**Figure 3.**
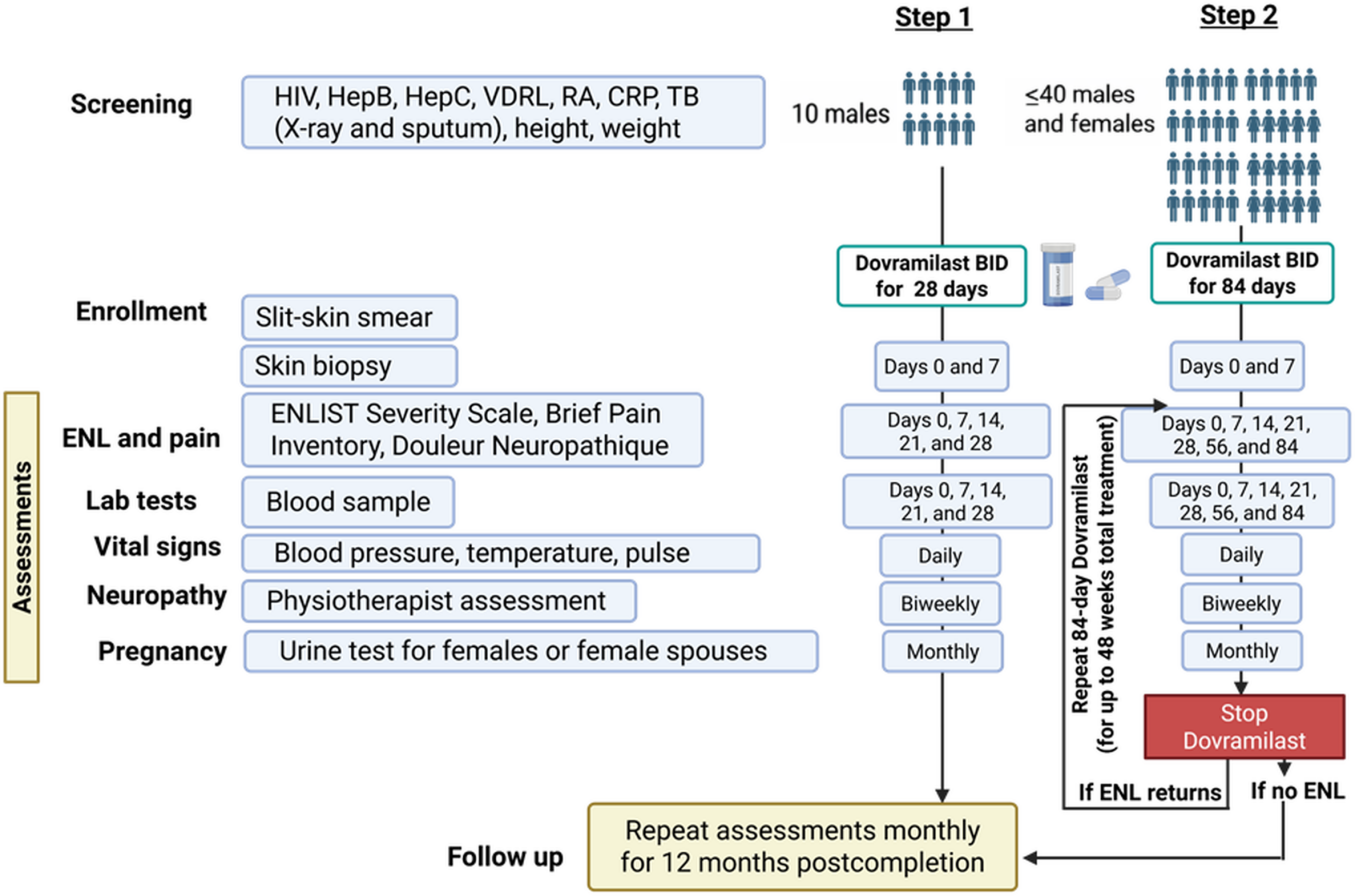
Overall study design showing treatment decision process and treatment durations. CRP: C-reactive protein; ENL: erythema nodosum leprosum; ENLIST: Erythema Nodosum Leprosum International Study Group; HepB: hepatitis B; HepC: hepatitis C; RA: rheumatoid arthritis; TB: tuberculosis; VDRL: Venereal Disease Research Laboratory test for syphilis. Created in BioRender [26].

This study will be a single-center, single-arm, phase 2, open-label trial to evaluate the safety and efficacy of 200 mg dovramilast administered twice daily and consumed with fatty food in participants with moderate-to-severe ENL. As outlined in Figure 3, the study will be performed in 2 steps.

- Step 1: 10 male participants with new or new episode ENL.
- Step 2: Up to 40 participants, including male and female patients with new or new episode ENL.

The primary aims of step 1 and step 2, respectively, are as follows:

1. To evaluate the immediate effect on the safety and efficacy of 28 days (1 mo) of dovramilast treatment in 10 males with a new or recurrent episode of ENL
2. To evaluate a 12-week treatment cycle of dovramilast, with the provision for further 12-week treatment cycles of dovramilast for up to 48 weeks (12 mo), in up to 40 participants, including males and females presenting with new or recurrent ENL

**Figure 4.**
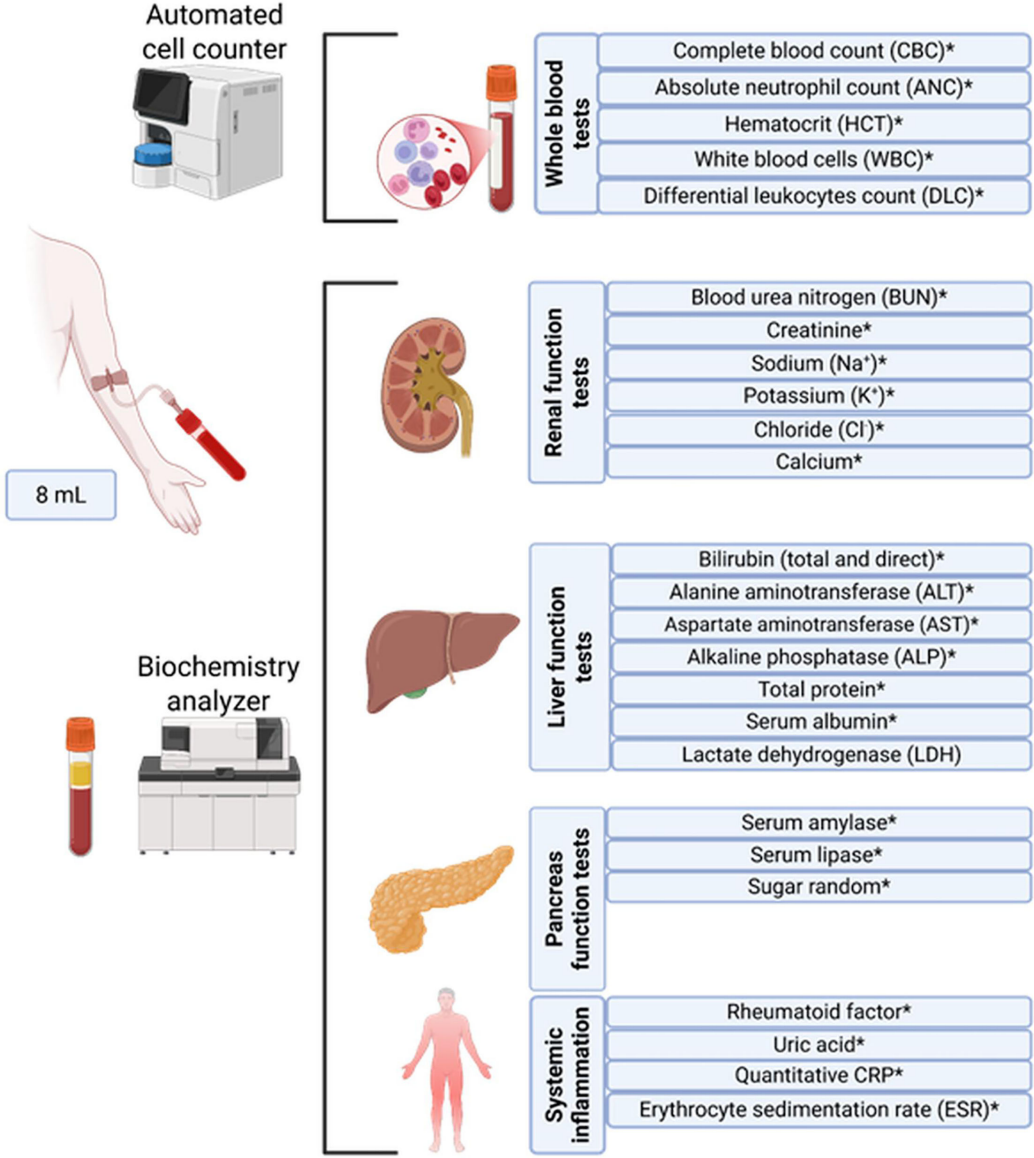
List of laboratory tests to be performed during the trial. The asterisk (*) indicates the subset of tests performed in step 1. All tests will be performed in step 2. Created in BioRender [26].

A safety analysis will be conducted on all participants who received at least 1 dose of the study drug and will include the frequency of all AEs and laboratory abnormalities (Figure 4), as well as the frequency of dose interruptions, dose reductions, and treatment discontinuations.

Study participants will be eligible for any concomitant treatment, including, but not limited to, WHO-recommended MDT, antipyretics, and analgesics. No standard ENL treatment regimens of prednisolone or thalidomide, or both, will be provided during the study drug treatment. In step 1, any new ENL episodes after the cessation of the study drug will be managed with standard ENL care. In step 2, any new ENL episodes will be managed with a repeat 12-week treatment cycle with dovramilast.

#### Step 1: Evaluation of the Immediate Effects

To evaluate the immediate effects of dovramilast, 10 male participants with moderate to severe, new, or recurrent ENL will receive an initial 10 days of treatment with 200 mg of dovramilast twice daily. The physician has the discretion to determine whether individual prospective participants with severe neuropathy or other severe symptoms should be enrolled. Participants will be males aged 18 to 65 years and weigh greater than or equal to 40 kg. The initial 28-day safety evaluation (step 1) was limited to male participants as a precautionary measure to minimize potential reproductive and pregnancy-related risks during early exposure to dovramilast in patients with ENL. Although dovramilast has demonstrated an acceptable safety profile in previous phase 1 and phase 2 studies in other disease settings, comprehensive reproductive safety data in ENL populations are limited. Restricting early enrollment to males allowed close monitoring of short-term safety and tolerability before broader inclusion. Upon confirmation of acceptable safety by the Data Safety Monitoring Board (DSMB), enrollment was expanded in step 2 to include both male and female participants, with appropriate exclusion of pregnant or breastfeeding women. Safety and efficacy monitoring assessments will be performed by DSMB representative clinicians during the initial 7- to 10-day treatment period. If assessments during the 10-day initial treatment trial indicate poor safety or efficacy, participants may be removed from the treatment no later than day 10. If assessments during the 10-day initial treatment period indicate that the drug is well tolerated and the participant’s ENL symptoms are responding, the treatment will be continued until day 28. If the drug treatment is not well tolerated, or if assessments for efficacy fail to demonstrate ENL symptom relief, participants may be removed from the treatment.

Upon review of the 10- and 28-day assessments, the DSMB will recommend that another cohort of 40 participants be enrolled for up to 12 months of treatment (step 2).

#### Step 2: Evaluation of the Long-Term Effects

Participants will be either male or female, aged 18 to 65 years, with a new or newly recurrent ENL episode and will receive 200 mg of dovramilast twice daily. Male weights will be aligned with step 1 requirements (≥40 kg), whereas females should weigh at least 35 kg.

In a subset of participants during step 2, pharmacokinetic assessments of drug levels in the blood will be performed using plasma collected at appropriate times on day 1 and day 10 (predose and 3 hours postdose) after dovramilast treatment.

As in step 1, participants will be enrolled for an initial 7 to 10 days of treatment. If patients tolerate the drug well and respond, they will continue to receive dovramilast for 12 weeks.

Every 12 weeks of treatment, dovramilast will be discontinued to determine if ENL symptoms recur or if the ENL episode has fully recovered. If symptoms recur after 28 days within the 1-year follow-up period, dovramilast will be administered again for another 12 weeks, as indicated by individual patient needs, for up to 1 year of total dovramilast treatment duration (12 wk × 4=48 wk total). Based on individual patient clinical needs, this discontinuation test will be repeated at 6 and 9 months for a total treatment period of up to 52 weeks. While clinical care may occur more frequently as indicated by individual ENL symptoms, participants will be evaluated on days 10 and 28 and monthly during treatment, and monthly for 1 year after discontinuation of the drug (final visit).

### Inclusion Criteria

Study participants must satisfy the following criteria for enrollment in the study:

1. Must be 18 to 65 years old, weighing >35 kg for women and >40 kg for men.
2. In step 1, the participants must be male. In step 2, the participants can be either male or female.
3. Understand and voluntarily sign an informed consent document prior to any study-related assessment or procedure.
4. Have signs or symptoms of new episodes of ENL.
5. Able to adhere to the study schedule and other protocol requirements. Patients with stable and well-managed diabetes and hypertension can be included in the study.
6. Women of childbearing age can be enrolled in step 2 following confirmation of safety in the initial male-only cohort, provided they are not pregnant or breastfeeding, have a negative pregnancy test at screening, comply with protocol-specified pregnancy testing, and agree to pregnancy prevention counseling (including abstinence where contraception is not culturally acceptable) during treatment and for 4 weeks after the last dose of study medication.

### Exclusion Criteria

The presence of any of the following will exclude a potential participant from enrollment:

1. Any significant medical condition, laboratory abnormality, or psychiatric illness that would prevent the participant from participating in the study.
2. Daily rifampicin treatment will be excluded with a minimum washout period of 2 weeks prior to dovramilast administration. However, if rifampicin is taken only once a month as part of MDT, this will not be used as an exclusion criterion.
3. Any condition, including the presence of laboratory abnormalities, that places the participant at an unacceptable risk if they were to participate in the study.
4. Any condition that confounds the ability to interpret data from the study (ie, HIV, chronic hepatitis B, chronic hepatitis C, or tuberculosis in patients under active treatment). Patients with resolved tuberculosis will not be excluded from the study.
5. Use of systemic corticosteroids or thalidomide within 7 days of study medication initiation.
6. Pregnant or nursing females.

### Primary and Secondary Outcomes

The primary outcome is the proportion of participants achieving a complete clinical response by day 10 of dovramilast treatment. Complete clinical response is defined as a clinically meaningful improvement or resolution of ENL manifestations based on standardized clinical criteria rather than a binary presence or absence. This includes a reduction in the number, tenderness, and inflammation of ENL skin nodules, assessed clinically and supported by changes in the EESS score; resolution of ENL-associated fever based on clinical assessment and temperature recordings; and improvement or resolution of nerve pain and tenderness evaluated through neurological examination and validated pain assessment tools. Safety outcomes include the frequency and severity of AEs, graded according to the National Cancer Institute Common Terminology Criteria for Adverse Events (NCI CTCAE) version 5.0 [71], as well as laboratory abnormalities and neurological assessments. In addition, plasma and skin biopsy samples will be collected at predefined time points for exploratory evaluation of biological responses to dovramilast, including inflammatory and immunological markers relevant to ENL pathogenesis (such as circulating cytokines, acute-phase reactants, and immune cell–associated markers), and histopathological features of ENL lesions. These exploratory analyses will be descriptive and are intended to provide mechanistic insights into treatment response.

Secondary outcomes include the proportion of participants who achieve and maintain remission of ENL symptoms during treatment and for up to 12 months following cessation of the study drug. Longitudinal changes in disease severity, pain, and neurological symptoms will be assessed over time using validated instruments, including the EESS, Brief Pain Inventory, and Douleur Neuropathique 4 questionnaire. In addition, the frequency of ENL recurrence and the need for repeat dovramilast treatment cycles during the follow-up period will be documented.

### Discontinuation of Treatment or Removal From the Study

A participant can be discontinued from ongoing drug treatment upon the occurrence of AEs related or unrelated to the drug treatment. If a participant fails to return for 1 or more follow-up appointments, data may still be collected upon attendance of the remaining appointments for up to 1 year after the last date of drug administration.

### Interventional Methods

Step 1: twice-daily administration of 200 mg of dovramilast to be taken with fatty food. The treatment will be for 10 days, with a rollover for a total of 28 days if safety and efficacy demonstrate benefit by day 10 for the patient. Step 2: twice-daily administration of 200 mg of dovramilast in patients to be taken with fatty food for 12 weeks, with additional 12-week treatment cycles (week 48) depending on individual clinical needs.

### Safety and Efficacy

Primary and secondary end points for efficacy, as explained above, will be assessed on day 0, day 28, and 1 year after study drug cessation. The severity of any toxicity will be graded according to version 5.0 of the NCI CTCAE. Summaries of demographics, disposition, study drug exposure, protocol violations, and AEs will also be made.

An independent DSMB oversees trial conduct and participant safety. The DSMB reviewed safety data from the initial safety cohort (step 1) prior to progression to the expanded treatment phase (step 2). Ongoing safety oversight includes periodic review of AEs, protocol deviations, and overall study conduct. Study monitoring is conducted in accordance with Good Clinical Practice and NHRC ethical guidelines to ensure data quality, protocol adherence, and participant protection.

### Data Analysis

This study is designed as an exploratory phase 2 pilot trial; therefore, analyses will be primarily descriptive and longitudinal rather than inferential. All analyses will be conducted according to the intention-to-treat principle, including all participants who receive at least 1 dose of dovramilast, regardless of treatment duration, protocol deviations, or withdrawal.

Primary outcome analysis will focus on estimating the proportion of participants achieving a complete clinical response by day 10 of treatment, based on predefined clinical criteria encompassing skin, systemic, and neurological manifestations of ENL. This proportion will be summarized with descriptive statistics and corresponding confidence intervals to provide an estimate of response frequency and variability. Secondary outcomes will be assessed through longitudinal evaluation of ENL severity, pain, and neurological symptoms using validated instruments (EESS, Brief Pain Inventory, and Douleur Neuropathique 4). Changes in these measures over time will be summarized descriptively using means, medians, ranges, and graphical trend displays to assess patterns of improvement, remission, and recurrence during treatment and follow-up. The frequency and timing of ENL recurrences and the need for repeat dovramilast treatment cycles will be documented to characterize treatment durability. Exploratory laboratory and tissue outcomes derived from plasma and skin biopsy samples will be analyzed descriptively to evaluate trends in inflammatory, immunological, and histopathological markers over time.

In addition to clinical and exploratory outcomes, feasibility will be assessed as a key objective of this pilot study. Feasibility indicators include the ability to recruit and retain participants according to the protocol, adherence to study medication and follow-up schedules, completeness of clinical and patient-reported outcome data, and acceptability of study procedures. These feasibility measures, together with observed variability in outcomes and safety findings, will be used to inform decisions regarding the design, operational requirements, and scalability of a future adequately powered randomized controlled trial.

Given the pilot nature of the study, missing data will not be imputed; analyses will be based on available data, and the extent and reasons for missing data will be described and considered in interpretation.

### Ethical Considerations

Ethical approval was obtained from the Government of Nepal, Nepal Health Research Council (NHRC reference number 409 for step 1 and 1485 for step 2) and the Department of Drug Administration (DDA reference number 2802 for step 1 and 58,900 for step 2). This study’s protocol adheres to all relevant ethical guidelines for research involving human participants.

Informed consent will be obtained prior to any study-related procedures. During the consent process, participants will receive comprehensive information about this study, including its objectives, procedures, potential benefits, travel fares, and any associated risks or discomforts. Participants will also be informed of their rights, including the option to withdraw from the study at any point, ensuring that participation is entirely voluntary.

Participant safety is a primary objective of this study. Potential harms associated with dovramilast are assessed through regular clinical evaluations, laboratory monitoring, neurological assessments, and systematic AE reporting. All AEs are graded using the NCI CTCAE version 5.0. Protocol-defined measures, including dose interruption, discontinuation, or withdrawal, are implemented in the event of clinically significant AEs. Clinical trial insurance was secured for all enrolled participants to cover compensation for any study-related injury or AEs, in compliance with NHRC ethical and regulatory guidelines.

To safeguard the privacy of participants, all collected data will be anonymized by assigning each participant a unique study number, ensuring that no personal identifiers are directly associated with the data. Demographic, clinical, and personal details will be securely stored in locked cabinets for all hard copies of the case report forms, as well as in a password-protected REDCap (Research Electronic Data Capture; Vanderbilt University) database. Only authorized research staff will have access to the data, and all information gathered during this study will be used exclusively for research purposes.

## Results

The initial safety evaluation (step 1), approved by the IRB on August 16, 2017, was completed in 2020 with enrollment of 10 male participants presenting with new or recurrent moderate-to-severe ENL. Review by the DSMB did not identify safety concerns that would preclude progression of the study, supporting continuation to the expanded treatment phase (step 2), which received IRB approval on February 15, 2024.

Based on findings from step 1, no substantive modifications to the study design, dosing regimen, or safety monitoring procedures were required. Step 2 recruitment procedures were initiated in September 2024, with enrollment planned to continue according to the protocol-specified sample size and follow-up schedule. All data are anticipated to be collected by September 2027, and the main results of the trial are anticipated to be published by December 2027. As of April 2026, a total of 15 participants have been recruited.

Recruitment, retention, and follow-up procedures have been implemented as outlined in the protocol. Any protocol deviations identified were documented and addressed according to Good Clinical Practice guidelines and did not necessitate protocol amendments.

Detailed analyses of safety, efficacy, and exploratory outcomes will be reported in a future results publication upon study completion.

## Discussion

### Principal Findings

This phase 2 study is designed to evaluate the safety, tolerability, and preliminary clinical effects of dovramilast in patients with new or recurrent ENL. We hypothesize that dovramilast will demonstrate an acceptable safety profile and be associated with clinically meaningful reductions in ENL severity and symptom burden, while reducing reliance on prolonged corticosteroid or thalidomide therapy. If confirmed, these findings would support further evaluation of dovramilast as a novel immunomodulatory treatment option for ENL.

### Comparison to Prior Work

Phosphodiesterase 4 (PDE4) inhibition has emerged as a potential immunomodulatory strategy for inflammatory diseases [72]. Apremilast, an oral PDE4 inhibitor approved for psoriasis and psoriatic arthritis [73], has been reported in limited case series and clinical experiences as a treatment option for ENL, particularly in patients with recurrent or steroid-dependent disease [74-76]. These reports suggest that PDE4 inhibition may reduce inflammatory burden and ENL symptoms while avoiding some of the long-term toxicities associated with corticosteroids and thalidomide.

However, evidence supporting apremilast use in ENL is scarce, highlighting the need for prospective clinical evaluation of PDE4 inhibitors in this setting. Dovramilast, a chemically distinct PDE4 inhibitor with demonstrated immunomodulatory effects and a favorable safety profile in other inflammatory and infectious disease studies, represents a rational candidate for systematic investigation in ENL through a structured phase 2 clinical trial.

### Strengths and Limitations

Strengths of this study include the use of validated ENL severity and pain scales, longitudinal follow-up for recurrence, and a staged design with independent safety oversight. Limitations include the open-label, single-arm design and inadequate sample size, which preclude formal efficacy comparisons but are appropriate for an exploratory phase 2 protocol.

### Future Directions

Findings from this study will inform the design of future randomized controlled trials, including the selection of primary end points, sample size estimation, and treatment duration. If dovramilast demonstrates sustained clinical benefit with acceptable safety, it may represent a steroid-sparing option for ENL, with important implications for long-term disability prevention in leprosy-affected populations.

### Dissemination Plan

Study findings will be disseminated through peer-reviewed publications, conference presentations, and reporting to relevant health authorities. Results will also be shared with participating clinicians and, where appropriate, with study participants and patient advocacy groups to support evidence-based management of ENL.

### Conclusions

This protocol describes an exploratory phase 2 study evaluating dovramilast for the treatment of new or recurrent ENL. The study is designed to assess safety, tolerability, feasibility, and preliminary clinical signals rather than to determine definitive efficacy. The anticipated findings will support the refinement of outcome measures, sample size estimates, and trial design for future randomized controlled studies. As such, this work represents an important step toward, but not yet evidence for, the broader clinical application of dovramilast in ENL management.

## Supplementary material

10.2196/88069 Checklist 1 SPIRIT checklist.

## Abbreviations

AE: adverse events
BL: borderline lepromatous
DSMB: Data and Safety Monitoring Board
EESS: ENL International Study Group ENL Severity Scale
ENL: erythema nodosum leprosum
IL: interleukin
LL: lepromatous leprosy
MDT: multidrug therapy
NCI CTCAE: National Cancer Institute Common Terminology Criteria for Adverse Events
PDE4: phosphodiesterase 4
REDCap: Research Electronic Data Capture
SPIRIT: Standard Protocol Items Recommendations for Interventional Trials
T1R: type 1 reaction
TNF: tumor necrosis factor
WHO: World Health Organization
